# Growth hormone pathways signaling for cell proliferation and survival in hippocampal neural precursors from postnatal mice

**DOI:** 10.1186/1471-2202-15-100

**Published:** 2014-08-26

**Authors:** Pablo Devesa, Fabienne Agasse, Sara Xapelli, Cristina Almengló, Jesús Devesa, Joao O Malva, Víctor M Arce

**Affiliations:** Department of Physiology, School of Medicine, University of Santiago de Compostela, 15710 Santiago de Compostela, Spain; Neuroprotection and Neurogenesis in Brain Repair Group, Center for Neuroscience and Cell Biology, School of Medicine, University of Coimbra, 3004-517 Coimbra, Portugal; Medical Center Proyecto Foltra, Travesía de Montouto 24, 15886 Teo, Spain

**Keywords:** GH, Neurogenesis, Apoptosis, Brain injury, Akt-mTOR, JNK

## Abstract

**Background:**

Accumulating evidence suggests that growth hormone (GH) may play a major role in the regulation of postnatal neurogenesis, thus supporting the possibility that it may be also involved in promoting brain repair after brain injury. In order to gain further insight on this possibility, in this study we have investigated the pathways signaling the effect of GH treatment on the proliferation and survival of hippocampal subgranular zone (SGZ)-derived neurospheres.

**Results:**

Our results demonstrate that GH treatment promotes both proliferation and survival of SGZ neurospheres. By using specific chemical inhibitors we have been also able to demonstrate that GH treatment promotes the activation of both Akt-mTOR and JNK signaling pathways, while blockade of these pathways either reduces or abolishes the GH effects. In contrast, no effect of GH on the activation of the Ras-ERK pathway was observed after GH treatment, despite blockade of this signaling path also resulted in a significant reduction of GH effects. Interestingly, SGZ cells were also capable of producing GH, and blockade of endogenous GH also resulted in a decrease in the proliferation and survival of SGZ neurospheres.

**Conclusions:**

Altogether, our findings suggest that GH treatment may promote the proliferation and survival of neural progenitors. This effect may be elicited by cooperating with locally-produced GH in order to increase the response of neural progenitors to adequate stimuli. On this view, the possibility of using GH treatment to promote neurogenesis and cell survival in some acquired neural injuries may be envisaged.

## Background

Since the discovery of newly generated neurons in the fully developed mammalian brain [1], research on adult neurogenesis has become one of the major goals of neuroscience. Under unperturbed conditions, postnatal neurogenesis is particularly important in two well-defined anatomical regions of the central nervous system (CNS): the subventricular zone (SVZ) lining the lateral walls of the anterior lateral ventricles; and the subgranular zone (SGZ) of the dentate gyrus (DG) of the hippocampus [2–5]. The neural stem cells (NSCs) stored within these neurogenic niches continuously self-renew and differentiate into multiple neural lineages, thus allowing the equilibrium between cell loss and cell replacement while maintaining the NSC pool [6]. In addition, accumulating evidence indicates that increased postnatal neurogenesis may be also observed in response to acute injury to the CNS, thus suggesting that new neurons may be generated in order to replace those lost owing to tissue wound and therefore contribute to the healing [7–11].

However, regardless of the time elapsed since the first discovery of postnatal mammalian neurogenesis, key information is still lacking about the regulatory mechanisms controlling the self-renewal and differentiation processes [12]. Postnatal neurogenesis has been shown to be regulated by a number of extrinsic factors such as pharmacological stimuli, an enriched environment or physical exercise [2, 13, 14]. In addition, postnatal neurogenesis is regulated by numerous hormones, among which the growth hormone (GH)-insulin-like growth factor-1 (IGF1) system seems to play a pivotal role [15]. The existence of important neurogenic (and neurotrophic) actions of GH in the adult brain has been demonstrated both in humans and laboratory animals [16–21]. Furthermore, GH has been shown to potentiate the neurogenesis induced in response to brain injury [22–24], thus leading to the possibility of using the hormone in the treatment of some neural diseases. However, little is known about the mechanisms underlying the neurogenic actions of GH, although they may depend, at least in part, on the stimulation of the proliferation, differentiation and survival of neural precursors. Therefore, this study was designed to investigate to role of GH in the regulation of the proliferation and survival of cultured neurospheres obtained from the DG of postnatal mice, as well as the molecular mechanisms underlying those effects.

## Results

### Establishment and characterization of SGZ-derived neurosphere cultures

**Figure 1:**
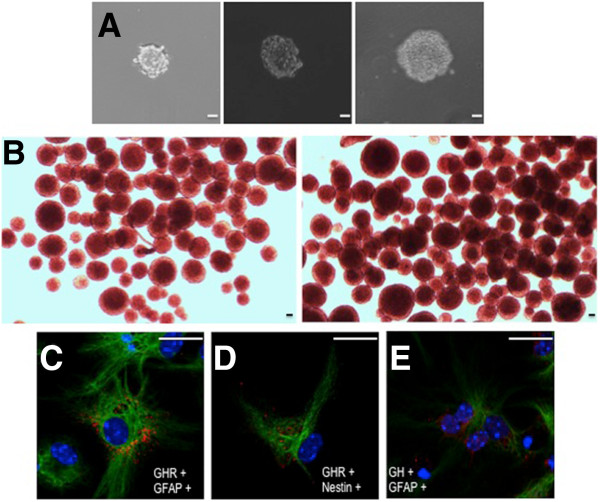
**GH and GHR expression in SGZ-derived neurospheres. A)** Evolution of neurosphere size after 7 (left), 12 (middle) or 15 (right) days in culture. **B)** Evolution of neurosphere number after 7-9 (left) or 13-15 (right) days in culture. In both cases, neurospheres were cultured in DMEM containing 1% Gibco B27 supplement, EGF (10 ng/mL) and FGF2 (10 ng/mL). **C** to **E)** SGZ-derived neurospheres express GHR and GH. Neurospheres were cultured for 7 days in DMEM containing 1% Gibco B27 supplement, and GH, GHR, GFAP and nestin immunoreactivities were detected with specific antibodies. GHR immunoreactivity is shown in red **(C, D)**. GH immunoreactivity is shown in red **(E)**. GFAP immunoreactivity is shown in green **(C, E)**. Nestin immunoreactivity is shown in green **(D)**. Scale bar = 10 μm.

SGZ-derived neurospheres obtained from neonatal (9 day old) mice were able to grow and give rise to new neurospheres upon passaging, thus demonstrating their capacity of self-renewal (Figures 1A and B, see also Table 1). In addition, these neurospheres were able to generate either GFAP- nestin- or O4-positive cells upon differentiation (data not shown). By using a polyclonal anti-GHR antibody we found the presence of GHR immunoreactivity in cells co-expressing either Nestin (Figure 1C) or GFAP (Figure 1D). In contrast, we have been unable to find GHR immunoreactivity in O4-positive cells (data not shown). GH immunoreactivity was also observed in neurosphere cells co-expressing GFAP (Figure 1D).

**Table 1:** **Evolution of neurosphere size and number**

***A)*** **Days**	**7**	**12**	**15**
Neurosphere size (mm)	23.1 ± 0.8	32.3 ± 0.9*	35.1 ± 1.5*
***B)*** **Days**	**7-9**	**13-15**	
Neurosphere number	139.4 ± 6.4	331.2 ± 10.0º	

Cells were grown in defined media as described for the indicated times. * = *p* < 0.01 vs day 7; ° = *p* < 0.01 vs day 7-9.

### GH treatment promotes the proliferation and survival of SGZ-derived neurospheres

**Figure 2:**
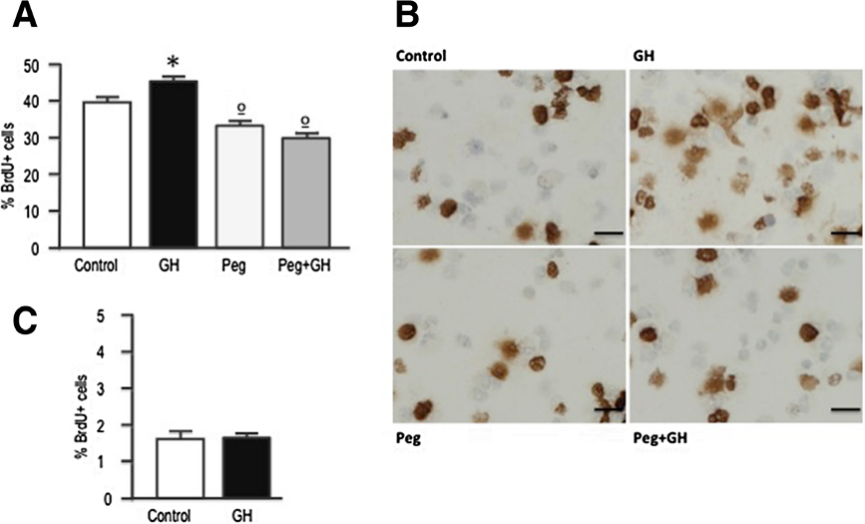
**GH treatment promotes the proliferation of SGZ neurospheres. A)** Neurospheres growing in defined media were treated for 24 h with GH (500 ng/mL), pegvisomant (Peg, 20 μg/mL), or GH + pegvisomant. Control cells were treated with saline. Four hours before the end of the treatment period cells were given a BrdU pulse (10 μM). Neurospheres were then dissociated, cells were collected by centrifugation onto coated cover slips, and BrdU was detected by immunocytochemistry. Each bar represents the mean + SEM of 3 experiments in triplicate. * = *p* < 0.05 *vs* Control; ° = *p* <0.05 *vs* Control and GH. **B)** Representative images of results presented in A. Scale bar = 10 μm. **C)** Neurospheres growing in defined media without EGF and FGF2 were treated for 24 h with GH or saline, and BrdU incorporation was detected as indicated. Each bar represents the mean + SEM of 3 experiments in triplicate.

Treatment of SGZ-derived neurospheres with GH resulted in a significant increase in the amount of BrdU incorporation (Figures 2A and B); an effect that was prevented by treating the cells with the GHR antagonist pegvisomant. Since pegvisomant hinders the binding of GH to its receptor, this finding demonstrates that GH-induced proliferation depends on stimulation of GHR. Interestingly, a significant decline in the proliferation of neurosphere cultures was also observed when pegvisomant was given alone (i.e. in the absence of exogenous GH) (Figures 2A and B). In contrast with these findings, GH was ineffective in promoting neurosphere proliferation when was given in the absence of EGF and FGF (Figure 2C).

**Figure 3:**
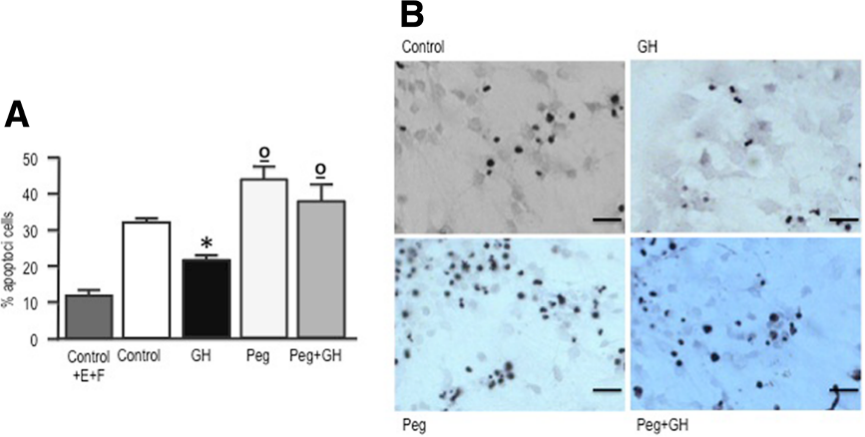
**GH treatment prevents apoptosis in SGZ neurospheres. A)** Neurospheres growing in defined media were placed in defined media without EGF and FGF2, and 24 h later treated with GH (500 ng/mL), pegvisomant (20 μg/mL), or GH + pegvisomant for an additional 48 h period. Control cells were treated with saline. Cell apoptosis was determined by TUNEL staining. Each bar represents the mean + SEM of 3 experiments in triplicate. * = *p* <0.05 *vs* Control; ° = *p* <0.05 *vs* Control and *p* <0.01 *vs* GH. Basal apoptosis was determined by growing the cells in the defined media (Control + EGF + FGF2). Apoptosis values in this group were significantly lower (*p* <0.01) than in the other groups. E = EGF; F = FGF2. **B)** Representative images of results presented in A. Scale bar = 10 μm.

In addition to promote proliferation, GH treatment was able to significantly reduce the apoptosis in neurosphere cultures (Figure 3). In order to induce cell death, neurosphere cultures were placed in media devoid of EGF and FGF. Under these experimental conditions, the number of apoptotic cells significantly increased with regard to cells growing in defined media (Figure 3), but this was counteracted by treating the cells with GH (Figure 3). Also in this case, the GH effect was prevented by treating the cells with pegvisomant, thus demonstrating the involvement of GHR. In concordance with the effect of pegvisomant on cell proliferation, a significant increase in apoptotic cell death was also observed when pegvisomant was given alone (Figure 3).

### Role of the Ras-ERK signaling pathway in the proliferative and antiapoptotic effects of GH

**Figure 4:**
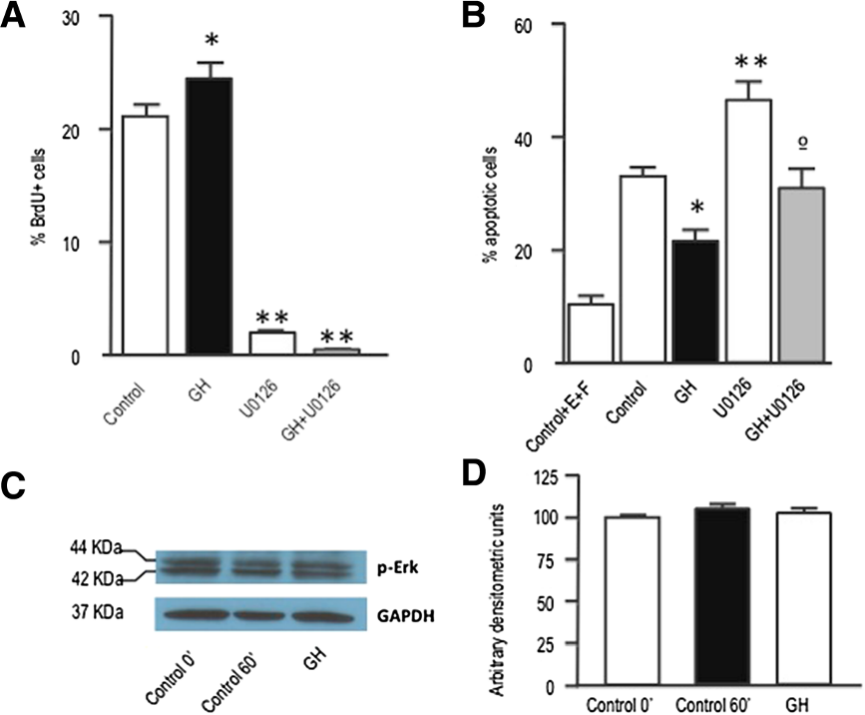
**ERK phosphorylation is necessary for neurosphere proliferation and survival. A)** Neurospheres growing in defined media were treated for 24 h with GH (500 ng/mL), U0126 (20 μM) or GH + U0126. Control cells were treated with saline. Four hours before the end of the treatment period cells were given a BrdU pulse (10 μM). Neurospheres were then dissociated and cells were collected by centrifugation onto coated cover slips, and BrdU was detected by immunocytochemistry. * = *p* <0.05 *vs* Control; ** = *p* < 0.001 *vs* control and GH. **B)** Neurospheres were placed into culture plates containing defined media without EGF and FGF2, and 24 h later treated with GH, U0126 or GH + U0126, for an additional 48 h period. Control cells were treated with saline. Cell apoptosis was determined by TUNEL staining. Each bar represents the mean + SEM of 3 experiments in triplicate. * = *p* <0.01 *vs* Control; ** = *p* <0.001 *vs* control and GH; ° = *p* <0.01 *vs* GH. Basal apoptosis was determined by growing the cells in the defined media (Control + EGF + FGF2). Apoptosis values in this group were significantly lower (*p* <0.01) than in the other groups. E = EGF; F = FGF2. **C)** Neurospheres were deposited for 48 h in slides with defined media without EGF and FGF2, and then treated with GH for 1 h. Phospho ERK and GAPDH immunoreactivities were determined by western blot. **D)** Densitometric evaluation of results presented in C. Phospho ERK levels are expressed as arbitrary densitometric units and normalized to GAPDH levels.

The importance of ERK phosphorylation in transducing GH actions has been previously demonstrated in different cell types [25, 26]. Therefore, in order to investigate the involvement of this signaling pathway in SGZ-derived neurosphere cultures, we treated them with U0126, a highly specific inhibitor of MEK1/2 that effectively blocks ERK phosphorylation. Blockade of Ras-ERK signaling not only resulted in a prominent inhibition of neurosphere proliferation (Figure 4A), but completely blocked the positive effect of GH on cell proliferation. Similarly, U0126 treatment induced a significant increase in the number of apoptotic cells, although in this case, the effect was partially counteracted by GH treatment (Figure 4B). Surprisingly, and in contrast with previous reports [25, 26], we have been unable to find any effect of GH on ERK phosphorylation in cultured SGZ neurospheres (Figures 4C and D).

### Role of the Phosphoinositide 3-kinase (PI-3 K)/Akt/mTOR signaling pathway in the proliferative and antiapoptotic effects of GH

**Figure 5:**
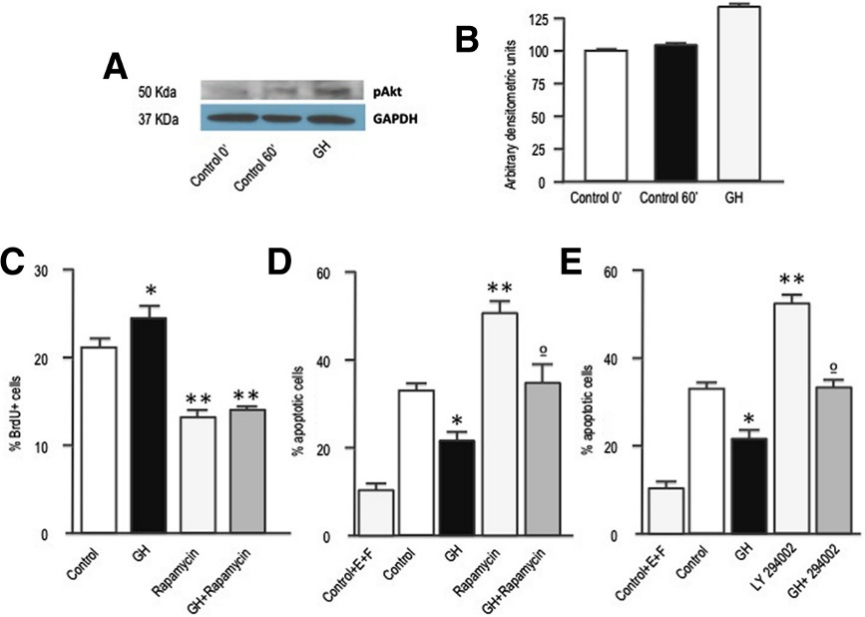
**The PI-3 K/Akt pathway is involved in the antiapoptotic affect of GH. A)** Neurospheres were deposited for 48 h in slides with defined media without EGF and FGF2, and then treated with GH for 1 h. Phospho Akt and GAPDH immunoreactivities were determined by western blot. **B)** Densitometric evaluation of results presented in A. Phospho Akt levels are expressed as arbitrary densitometric units and normalized to GAPDH levels. **C)** Neurospheres growing in defined media were treated for 24 h with saline (Control), GH (500 ng/mL), rapamycin (20 nM), or GH + rapamycin. Four hours before the end of the treatment period cells were given a BrdU pulse (10 μM). Neurospheres were then dissociated and cells were collected by centrifugation onto coated cover slips, and BrdU was detected by immunocytochemistry. * = *p* <0.05 *vs* Control; ** = *p* <0.001 *vs* Control and GH. **D)** and **E)** Neurospheres were placed into culture plates containing defined media without EGF and FGF2, and 24 h later treated for 48 h with saline, GH, rapamycin or GH + rapamycin, **[D)]** or saline, GH, LY 294002 (10 μM) or GH + LY 294002, **[E)]**. * = *p* < 0.01 vs Control; ** = *p* <0.01 *vs* Control and *p* <0.001 *vs* GH; ° = *p* <0.01 *vs* GH. In D) and E) basal apoptosis, determined by growing the cells in the defined media (Control +E+ F), was significantly lower (p <0.01) than in the other groups. E = EGF; F = FGF2. Bars = mean + SEM of 3 experiments in triplicate.

The role of the PI-3 K/Akt/mTOR pathway in GH signaling has been also supported by several previous observations [27, 28]. Activation of GHR induces the phosphorylation of the GHR-associated protein Insulin receptor substrate 1 (IRS1) which, in turn, activates PI-3 K that promotes Akt phosphorylation. In keeping with those findings, GH treatment induced a prompt and marked rise in phospho-Akt levels in SGZ-derived neurospheres (Figures 5A and B). Once phosphorylated, Akt activates the phosphoinositide-dependent kinase-1 (PDK1) that, in turns, phosphorylates a number of intracellular targets including mTOR [28] which, reportedly, plays a major role in the regulation of proliferation and survival in different types of cells [29, 30]. Therefore, in order to assess the involvement of the PI-3 K/Akt/mTOR pathway on GH actions, we first treated neurosphere cultures with rapamycin, an inhibitor of the mTOR complex 1 (mTORC1). As Figure 5C depicts, treatment with rapamycin significantly reduced the proliferation of neurosphere cultures, and completely abolished the effect of GH. Interestingly rapamycin also induced a significant increase in cell death but, in this case, the effect was partially counteracted by GH (Figure 5D). In order to further investigate the involvement on Akt stimulation of this effect, cells were also treated with LY294002, a potent inhibitor of PI-3 K. In keeping with previous results in other cell types [27, 31, 32], inhibition of Akt phosphorylation significantly increased the number of apoptotic cells (Figure 5E) while, as occurred with rapamycin treatment, GH was able to partially overcome this effect (Figure 5E).

### GH-induced JNK phosphorylation is involved in the antiapoptotic effects of GH

**Figure 6:**
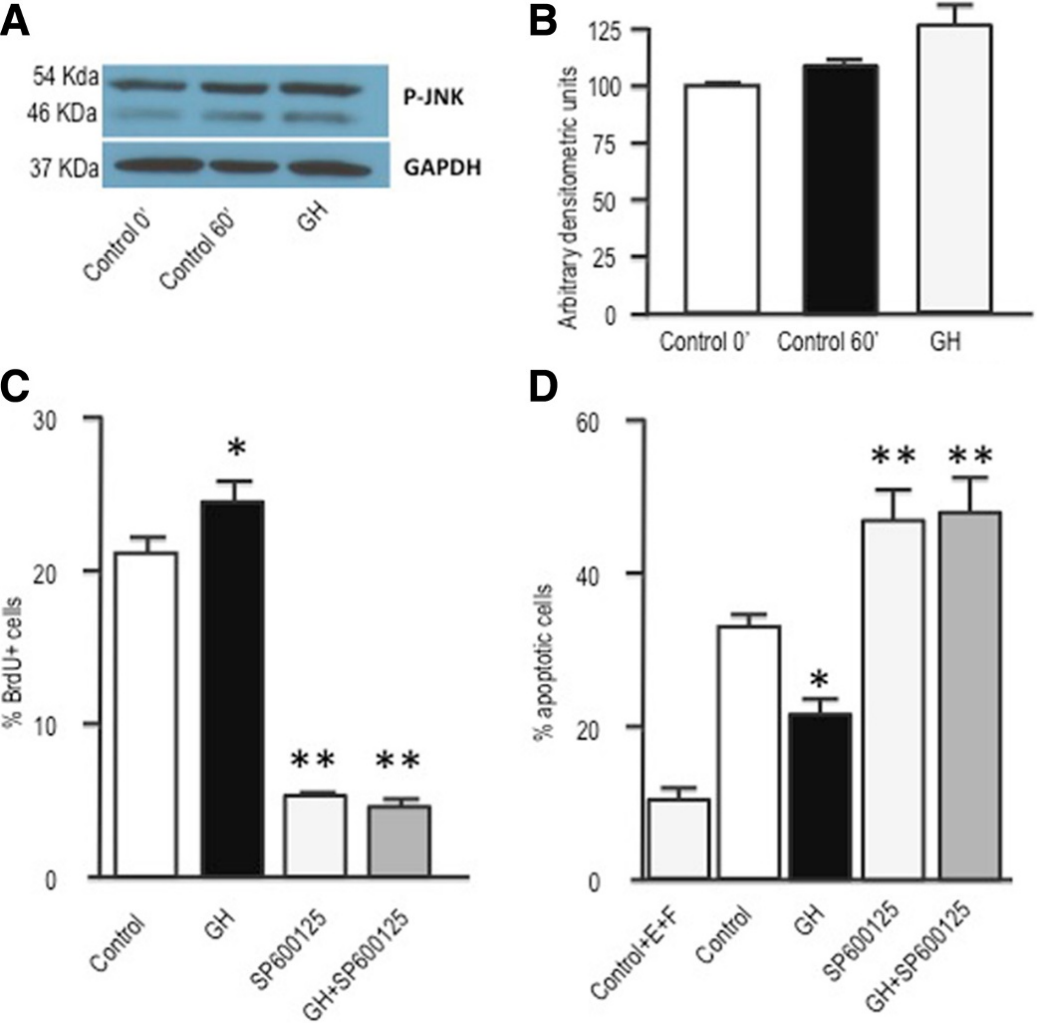
**JNK phosphorylation is involved in the proliferative and antiapoptotic affects of GH. A)** Neurospheres were deposited for 48 h in slides with defined media without EGF and FGF2, and then treated with GH for 1 h. Phospho JNK and GAPDH immunoreactivities were determined by western blot. **B)** Densitometric evaluation of results presented in A. Phospho JNK levels are expressed as arbitrary densitometric units and normalized to GAPDH levels. **C)** Neurospheres growing in defined media were treated for 24 h with GH (500 ng/mL), SP600125 (20 μM) or SP600125 + GH. Control cells were treated with saline. Four hours before the end of the treatment period cells were given a BrdU pulse (10 μM). Neurospheres were then dissociated and cells were collected by centrifugation onto coated cover slips, and BrdU was detected by immunocytochemistry. * = *p* <0.05 *vs* Control; ** = *p* <0.001 *vs* Control and GH. **D)** Neurospheres were placed into culture plates containing defined media without EGF and FGF2, and 24 h later treated with GH, SP600125 or SP600125 + GH, for an additional 48 h period. Basal apoptosis was determined by growing the cells in the defined media (Control + EGF + FGF2). Apoptosis values in this group were significantly lower (*p* <0.01) than in the other groups. * = *p* <0.05 *vs* Control; ** = *p* <0.001 *vs* Control and GH. E = EGF; F = FGF2. Bars= mean + SEM of 3 experiments in triplicate.

Finally, we investigated the role of JNK in mediating the effects of GH on neurosphere proliferation and survival. In keeping with previous findings in fibroblasts [31] or macrophages [32], GH was able to induce the phosphorylation of JNK in cultured neurospheres (Figures 6A and B). When JNK actions were counteracted by treating the cells with SP600125, a competitive inhibitor of its kinase activity, both proliferation and survival were significantly decreased in neurosphere cultures (Figures 6C and D). Interestingly, none of these effects could be counteracted by GH treatment.

## Discussion

This study was designed to investigate the role of GH on postnatal neurogenesis using a neurosphere culture system. The presence of ongoing neurogenesis in the postnatal mammalian brain, together with the prospect that the stem cell pools present in the adult brain can be directed towards repair of neural injuries has generated much interest for the last several years, see [19, 33] for review. Furthermore, since postnatal neurogenesis is regulated by a complex network of signaling molecules and extrinsic factors [12], understanding these mechanisms of control will help to find effective treatments directed towards increase neurogenesis in order to promote brain repair.

GH has been proposed to play a role on brain repair after injury [34]. Although some of the beneficial effects of GH on brain repair may depend on their neuroprotective actions [33], there is also accumulating evidence indicating that neurogenesis is also stimulated by GH. Thus GH treatment has been demonstrated to promote neurogenesis in different brain areas [20, 23, 24, 35] either under unperturbed conditions [20, 35] or in response to brain damage [23, 24]. Interestingly, GH-induced neurogenesis is potentiated when GH treatment is combined with physical exercise [35, 36] or physical rehabilitation [24], thus suggesting that the hormone is not only cooperating with endogenous mechanisms regulating postnatal neurogenesis, but may be even essential to induce the neurogenic response [36]. There is also evidence indicating that GH increases the frequency of both rodent- and human-derived neurospheres in vitro [37–41]; and the present study], thus demonstrating a direct effect of GH on neural precursor cells. Furthermore, in all these cases, GH actions are lost when GHR is absent or inactivated, thus indicating that GH actions are exerted via activation of GHR; although the possibility that some of GH actions on neural precursors may depend on the activation of other receptors cannot be ruled out [41].

Once established the importance of GH in the regulation of proliferation and survival of SGZ-derived neurospheres, we next investigated the molecular mechanism underlying these actions. The predominant GH signal transduction cascade comprises activation of the GHR dimer and phosphorylation of Janus kinase 2 (JAK2). Activated JAK2 then phosphorylates key tyrosine residues on the cytoplasmic domain of the GHR, permitting SH2 domain interactions by signal adaptor proteins that results in docking of signal transducer and activator of transcription 5 (STAT5) for activation by JAK2 [25, 26]. The triggering of this canonical signaling pathway has been recently demonstrated in neurosphere cells [38], and may be involved in some of the GH actions on these cells. However, phosphorylation of the cytoplasmic domain of the GHR also results in the activation of the Ras/MAPK kinase/ERK pathway; while activated JAK2 is also able to directly phosphorylate IRS-1/2, facilitating activation of the PI3K/Akt/mTOR pathway [25, 26].

Our findings demonstrate that at least two of those signaling pathways, the PI-3 K/Akt/mTOR and JNK module of the MAPK, are activated by GH in cultured neurospheres, thus illustrating the complexity of GHR signaling in these cells. Furthermore, by using selective inhibitors we have been able to demonstrate that, despite these signaling pathways may be important in mediating the GH effects on cell proliferation, they appear to exert a more essential role in the regulation of cell survival. There are several types of evidences supporting our hypothesis. First, GH was able to partially overcome the effect of ERK inhibition on cell survival, while its effect on cell proliferation was completely abrogated under these circumstances. The ability of GH to promote cell survival in the absence of ERK signaling indicates that GH is able to deliver a survival signal that is independent on Ras/ERK pathway activation. In contrast, Ras/ERK signaling is necessary for the GH effect on neurosphere proliferation indicating that, in keeping with previous reports [41, 42], GH is neither sufficient nor necessary for neurosphere proliferation.

Second, GH treatment also partially counteracted the negative effect of rapamycin on cell survival, but did not modify rapamycin-induced inhibition of cell proliferation. Interestingly, mTOR has been shown to be relevant for EGF-dependent proliferation of neural precursors [43], further supporting our hypothesis regarding a minor role of GH on the regulation of neurosphere proliferation. Furthermore, mTOR is present in two distinct complexes: mTOR complex 1 (mTORC1) and mTOR complex 2 (mTORC2) [30, 44]. Since only mTORC1 is inhibited by rapamycin, the possibility exists that GH actions on cell survival may depend, at least in part, on the rapamycin-insensitive mTORC2 complex, as occurs with insulin [45]. Another possible mechanism, not excluding the former, is that GH may overcome the effect of rapamycin treatment via the stimulation of survival signals that do not depend on the PI-3 K/Akt/mTOR pathway. In this regard, it is interesting to indicate that the ability of GH to prevent apoptosis was preserved in the presence of PI-3 K inhibition, thus suggesting that Akt activation does not play a major anti-apoptotic role in these cells.

In contrast with these findings, GH effects on both cell proliferation and survival were completely inhibited when the phosphorylation of JNK was blocked. JNKs are members of the MAPK family that are activated by a variety of environmental stresses, inflammatory cytokines and, in some instances, by growth factors and GPCR (G protein-coupled receptor) agonists [46]. Once activated, the JNK pathway regulates numerous cellular responses including cell proliferation and survival, together with different aspects of neural development [47–50]. Therefore, the fact that blockade of JNK signaling completely inhibited both the proliferation and survival of SVZ-derived neurospheres, indicates that this pathway plays a major role in the regulation of the cellular fate of these cells. Furthermore, the fact that both GH-induced proliferation and survival are also blocked under these experimental conditions, together with the ability of GH to promote JNK phosphorylation, indicates that the JNK pathway is a key component of the signaling machinery activated by GH in SVZ-derived neurospheres. To our knowledge, this is the first time to demonstrate the capacity of GH to induce the phosphorylation of JNK in neural cells. To the present, GH-induced JNK phosphorylation has been demonstrated in macrophages [34], in NIH 3 T3 fibroblasts [33], and also in chinese hamster ovary cells stably transfected with rat GH receptor cDNA [51]. However, GH has been shown to reduce both basal and doxorubicin-stimulated JNK transcriptional activity and phosphorylation in both MDA-MB-231 and MCF7 cells [52], thus demonstrating the complexity of GH-mediated JNK regulation.

Finally, it is noteworthy to indicate that blockade of GH signaling was able to induce a decrease in neurosphere proliferation and survival even in cells not receiving GH treatment. This finding, together with the existence of GH expression in cultured neurospheres ([41]; and the present report), suggests the existence of an endogenous GH axis regulating the proliferation and apoptosis of neural progenitors. Up to now, the existence of GH immunoreactivity has been reported in several brain areas that include germinal regions of the embryonic brain [53], as well as brain regions involved in postnatal neurogenesis [54–56]. Furthermore, GH gene expression within these areas is increased by factors known to increase neurogenesis such as learning, exercise or estrogen administration (37; 54, 55), while such increase is not observed in GHR-/- animals (37). Altogether, these findings lead to the possibility that GH treatment is, in fact, cooperating with locally-produced GH in increasing the proliferation of neural progenitors in response to adequate stimuli. In this regard, it is interesting to note that we have previously found that GH is expressed in rat hippocampal progenitors, and that GH expression increases after neurotoxic damage (23). Therefore, it is tempting to speculate that, this locally-produced GH may cooperate with the exogenous hormone in promoting neurogenesis and cell survival in response to brain injury.

## Conclusions

Altogether, these findings lead to the possibility that GH treatment is, in fact, cooperating with locally-produced GH in increasing the proliferation and survival of neural progenitors in response to adequate stimuli. In this regard, the fact that increased GH expression has been detected in hippocampal progenitors after neurotoxic damage [23] suggests that endogenous and exogenous GH may also cooperate in promoting neurogenesis and cell survival in response to brain injury.

## Methods

### SGZ cultures and treatments

All animal procedures were carried out in accordance with European Union guidelines for the care and use of laboratory animals and they were approved by the Ethics committees of the Universities of Santiago de Compostela and Coimbra. SGZ cells were obtained from 9-day-old C57BL/6 donor mice as previously described [25]. Briefly, brains were removed under sterile conditions and placed in calcium- and magnesium-free Hanks’ balanced salt solution (Life Technologies Corporation, Carlsbad, CA). Sagittal brain sections (350 μm-thick) prepared with a McIlwain tissue chopper were used to dissect SGZ fragments. Fragments were then digested in 0.025% trypsin (Life Technologies Corporation) and 0.265 mM EDTA (Life Technologies Corporation) for 10 minutes at 37°C, and gently dissociated with a 1000 mL plastic pipette tip. The resulting cell suspension was diluted in Dulbecco’s modified Eagle’s medium (DMEM) supplemented with 100 U/mL penicillin, 100 μg/mL streptomycin, 1% Gibco B27 supplement, 10 ng/mL epidermal growth factor (EGF), and 10 ng/mL basic fibroblast growth factor 2 (FGF2) (defined media; all from Life Technologies Corporation). Cells were then plated on uncoated dishes (Corning Incorporated, Corning, NY) at a density of 3,000 cells per cm^2^ and maintained at 37°C in a 5% CO_2_ atmosphere in order to allow neurosphere development. Ten to 12 days after plating, the SGZ-derived neurospheres were collected and placed in fresh DMEM and used for cell proliferation studies. In order to induce cell apoptosis, neurosphere cells were placed in DMEM supplemented with 100 U/mL penicillin, 100 μg/mL streptomycin and 1% Gibco B27 supplement. Further experimental details are provided in the corresponding figure legend.

Recombinant human GH (Humatrope) was obtained from Eli Lilly and Company (Madrid, Spain); pegvisomant (Somavert), an antagonist of GH receptor (GHR), was obtained from Pfizer (Madrid, Spain); the extracellular signal-regulated kinase (ERK) inhibitor U0126, the mammalian target of rapamycin (mTOR) inhibitor rapamycin, and the c-Jun N-terminal kinase (JNK) inhibitor SP600125 were purchased from Sigma-Aldrich (Madrid, Spain). The PI-3 K inhibitor LY 294002 was purchased from Promega (Madison, WI).

### Immunofluorescence

After fixation in 4% PFA for 30 minutes at room temperature, SGZ cells were permeabilized, and nonspecific binding sites were blocked with 0.25% Triton X-100 and 3% BSA for 30 minutes at room temperature. SGZ cells were then incubated overnight at 4°C with the following primary antibodies: polyclonal anti-GHR (Abcam, Cambridge, MA; dilution 1:500), polyclonal anti-GH (Abcam; dilution 1:200), monoclonal anti-glial fibrillary acidic protein (GFAP) (Cell Signaling Technology, Danvers, MA; dilution 1:500), monoclonal anti-nestin (EMD Millipore Corporation, Billerica, MA; dilution 1:200), monoclonal anti-O4 (EMD Millipore Corporation; dilution 1:200). Coverslips were then rinsed in PBS and incubated for 1 hour at room temperature with the appropriate secondary antibodies: anti-rabbit IgG labeled with Alexa Fluor 488 (1:200) or with Alexa Fluor 594 (1:200), or anti-mouse IgG labeled with Alexa Fluor 594 (1:200) (all from Life Technologies). After an additional rinse in PBS, cell nuclei were stained with Hoechst 33342 (Life Technologies, 2 μg/mL in PBS containing 0.25% BSA) for 5 minutes at room temperature. Finally, the preparations were mounted using Dako-Cytomation fluorescent medium (DakoCytomation, Carpinteria, CA). Negative controls without primary antibody application showed lack of specific immunoreactivity with minimum background staining (data not shown). Fluorescent images were recorded using a confocal microscope (LSM 510 Meta; Carl Zeiss, Gottingen, Germany) or an Axioskop 2 Plus fluorescent microscope (Carl Zeiss).

### Cell proliferation studies

To assess cell proliferation, neurosphere cells growing in defined media received a treatment for 24 hours and were exposed to 10 μM 5-bromo-2′-deoxyuridine (BrdU) (Sigma-Aldrich) for the last 4 hours. Cultures were then fixed in 4% PFA for 30 minutes and, after extensive rinsing (30 minutes in 0.15 M PBS, at room temperature), BrdU was unmasked by successive passages in: 1% Triton X-100 for 30 minutes at room temperature, ice-cold 0.1 M HCl for 20 minutes, and 2 M HCl for 40 minutes at 40°C. Following neutralization in sodium borate buffer (0.1 M Na_2_B_4_O_7_ · 10H_2_O, pH 8.5; Sigma-Aldrich) for 15 minutes at room temperature, slices were rinsed in PBS, and nonspecific binding sites were blocked with 3% bovine serum albumin (BSA; Sigma-Aldrich) and 0.3% Triton X-100 for 30 minutes at room temperature. SGZ cultures were then incubated overnight at 4°C with a primary anti-BrdU antibody (EMD Millipore Corporation; dilution 1:50) in PBS containing 0.1% Triton X-100 and 0.3% BSA. After rinsing in PBS, cells were incubated with a secondary antibody labeled with Alexa Fluor 594 (Life Technologies, dilution 1:200) for 1 hour at room temperature. After an additional rinse in PBS, cell nuclei were stained with Hoechst 33342 (Life Technologies, 2 μg/mL in PBS containing 0.25% BSA) for 5 minutes at room temperature. Finally, the preparations were mounted using DakoCytomation fluorescent medium (DakoCytomation). Fluorescent images were recorded using a confocal microscope (LSM 510 Meta; Carl Zeiss) or an Axioskop 2 Plus fluorescent microscope (Carl Zeiss). For each well, 8 random fields were photographed and BrdU + cells were scored.

### Cell survival studies

Cell apoptosis was evaluated by terminal deoxy-nucleotidyl transferase dUTP nick-end labeling (TUNEL), as previously described [57]. Briefly, permeabilized cells were incubated in terminal deoxy-nucleotidyl transferase buffer (0.25 U/μl terminal transferase, 6 μM biotinylated dUTP, pH 7.5; all from Roche Farma, S.A, Madrid, Spain) for 1 hour 30 minutes at 37°C in a humidified chamber. The enzymatic reaction was stopped by 15 minutes of incubation in 300 mM NaCl (Sigma-Aldrich) and 30 mM sodium citrate buffer (Sigma-Aldrich). Following an additional rinse in PBS, cultures were incubated for 30 minutes at room temperature with the avidin-biotin-peroxidase complex (1:100; Vector Laboratories, Burlingame, CA). Peroxidase activity was revealed by the 3,3′-diaminobenzidine chromogen (0.025%; Sigma-Aldrich) intensified with 0.08% NiCl_2_ in 30 mM Tris-HCl (pH 7.6) buffer containing 0.003% H_2_O_2_. The cell preparations were then dehydrated in ethanol (70%, 2 minutes; 80%, 2 minutes; 90%, 2 minutes; 95%, 2 minutes; 100%, 2 minutes), cleared in xylene (3 minutes), and mounted using DEPEX mounting medium (Sigma-Aldrich). Photomicrographs of TUNEL were recorded using a digital camera (Axiocam HRC; Carl Zeiss) adapted to an Axioskop 2 Plus fluorescent microscope (Carl Zeiss). For each well, 8 random fields were photographed and positive cells were scored.

### Western blot

For phospho-ERK, phospho-Akt and phospho-JNK determination, neurospheres were maintained for 12-13 days in DMEM supplemented with 100 U/mL penicillin, 100 μg/mL streptomycin, 1% Gibco B27 supplement, EGF (10 ng/mL), and FGF2 (10 ng/mL); and then placed in plates containing DMEM supplemented with 100 U/mL penicillin, 100 μg/mL streptomycin and 1% Gibco B27 supplement for 12 h. Finally, the medium was replaced with fresh DMEM supplemented with 100 U/mL penicillin, 100 μg/mL streptomycin and 0.1% Gibco B27 supplement, and incubated for an additional 12 h period. Cells were then collected by centrifugation and the pellet was then lysated by heating at 95°C for 5 min in 1% SDS, and immediately cooled at 4°C for 15 min with ice-cold lysis buffer (50 mM Hepes, pH 7.5; 150 mM NaCl; 10% glycerol; 1% triton X-100; 5 mM EGTA; 1.5 mM MgCl_2_; 20 mM Na_4_P_2_O_7_; 20 mM Na_3_VO_4_; 50 μg/mL aprotinin and 4 mM phenylmethylsulfonyl fluoride). After centrifugation (15,000 g, 15 min, 4°C) to separate cellular debris, the lysates were resolved in a 12% SDS-PAGE, and electrotransferred onto nitrocellulose paper (Protran; Schleicher and Schuell, Dassel, Germany). Primary antibodies rose against pAkt, pERK, pJNK and glyceraldehyde 3-phosphate dehydrogenase (GAPDH) (all from Cell Signaling, dilution 1:1000), were applied overnight at 4°C, and were detected using alkaline phosphatase-conjugated secondary antibodies (dilution 1:20000). Immunoreactive bands were detected with a western-light chemiluminescence detection system (ECL; APBiotech, Little Chalfont, UK) and photographed (HyperfilmECL; APBiotech). Immunoreactive bands were scanned with a GelDoc system (Bio-Rad, Hercules, Calif., USA) and optical density was determined with the ImageJ software (National Institutes of Health, Bethesda, MD, USA).

### Statistics

Statistical analysis was performed with the non-parametric Mann–Whitney test. Statistical significance was established at *P* < 0.05.

## Authors’ information

PD: PhD, researcher in Foundation Foltra. FA: PhD, researcher in the Neuroprotection and Neurogenesis in Brain Repair Group, Center for Neuroscience and Cell Biology, School of Medicine, University of Coimbra. SX: PhD, researcher in the Neuroprotection and Neurogenesis in Brain Repair Group, Center for Neuroscience and Cell Biology, School of Medicine, University of Coimbra. CA. Researcher in Foundation Foltra and PhD student at the University of Santiago de Compostela. JD: MD, PhD, Professor of Physiology, Scientific Director of Foundation Foltra, Professor *Ad Honorem* of the University of Santiago de Compostela. JOM: PhD, Head of the Neuroprotection and Neurogenesis in Brain Repair Group, Center for Neuroscience and Cell Biology, School of Medicine, University of Coimbra. VMA: MD, PhD, Professor of Physiology at the School of Medicine of the University of Santiago de Compostela.

## Abbreviations

GH: Growth hormone
GHR: Growth hormone receptor
CNS: Central nervous system
SGZ: Subgranular zone
DG: Dentate gyrus
NSC: Neural stem cells
IGF1: Insulin-like growth factor-1
GFAP: Glial fibrilar acidic protein
BrdU: 5-bromo-2′-deoxyuridine
ERK: Extracellular signal-regulated kinases
MEK1/2: Mitogen-activated protein kinase kinase
PI-3 K: Phosphoinositide -3kinase
Akt: Protein kinase B
mTOR: Mammalian target of rapamycine
IRS1: Insulin receptor substrate 1
PDK1: Phosphoinositide-dependent kinase-1
JNK: c-Jun N-teminal kinases
JAK2: Janus kinase 2
STAT5: Signal transducer and activator of transcription 5
MAPK: Mitogen activated protein kinase
mTORC1: Mammalian target of rapamycine complex 1
mTORC2: Mammalian target of rapamycine complex 2
GPCR: G protein-coupled receptor
EGF: Epidermal growth factor
FGF2: Fibroblast growth factor 2
TUNEL: Terminal deoxy-nucleotidyl transferase dUTP nick-end labeling.

## References

[1] Altman J, Das GD (1965). Autoradiographic and histological evidence of postnatal hippocampal neurogenesis in rats. J Comp Neurol.

[2] Kempermann G, Kuhn HG, Gage FH (1997). Genetic influence on neurogenesis in the dentate gyrus of adult mice. Proc Natl Acad Sci U S A.

[3] Gage FH (1998). Stem cells of the central nervous system. Curr Opin Neurobiol.

[4] Markakis EA, Gage FH (1999). Adult-generated neurons in the dentate gyrus send axonal projections to field CA3 and are surrounded by synaptic vesicles. J Comp Neurol.

[5] Zhao C, Deng W, Gage FH (2008). Mechanisms and functional implications of adult neurogenesis. Cell.

[6] Gage FH, Temple S (2013). Neural stem cells: generating and regenerating the brain. Neuron.

[7] Abdipranoto A, Wu S, Stayte S, Vissel B (2008). The role of neurogenesis in neurodegenerative diseases and its implications for therapeutic development. CNS Neurol Disord Drug Targets.

[8] Abdipranoto-Cowley A, Park JS, Croucher D, Daniel J, Henshall S, Galbraith S, Mervin K, Vissel B (2009). Activin A is essential for neurogenesis following neurodegeneration. Stem Cells.

[9] Liu J, Solway K, Messing RO, Sharp FR (1998). Increased neurogenesis in the dentate gyrus after transient global ischemia in gerbils. J Neurosci.

[10] Magavi SS, Leavitt BR, Macklis JD (2000). Induction of neurogenesis in the neocortex of adult mice. Nature.

[11] Parent JM, Yu TW, Leibowitz RT, Geschwind DH, Sloviter RS, Lowenstein DH (1997). Dentate granule cell neurogenesis is increased by seizures and contributes to aberrant network reorganization in the adult rat hippocampus. J Neurosci.

[12] Mu Y, Lee SW, Gage FH (2010). Signaling in adult neurogenesis. Curr Opin Neurobiol.

[13] van Praag H, Kempermann G, Gage FH (1999). Running increases cell proliferation and neurogenesis in the adult mouse dentate gyrus. Nat Neurosci.

[14] van Praag H, Schinder AF, Christie BR, Toni N, Palmer TD, Gage FH (2002). Functional neurogenesis in the adult hippocampus. Nature.

[15] Devesa J, Devesa P, Reimunde P (2010). Growth hormone revisited. Med Clin (Barc).

[16] Devesa J, Reimunde P, Devesa A, Souto S, Lopez-Amado M, Devesa P, Arce VM (2009). Recovery from neurological sequelae secondary to oncological brain surgery in an adult growth hormone-deficient patient after growth hormone treatment. J Rehabil Med.

[17] High WM, Briones-Galang M, Clark JA, Gilkison C, Mossberg KA, Zgaljardic DJ, Masel BE, Urban RJ (2010). Effect of growth hormone replacement therapy on cognition after traumatic brain injury. J Neurotrauma.

[18] Reimunde P, Quintana A, Castañón B, Casteleiro N, Vilarnovo Z, Otero A, Devesa A, Otero-Cepeda XL, Devesa J (2011). Effects of growth hormone (GH) replacement and cognitive rehabilitation in patients with cognitive disorders after traumatic brain injury. Brain Inj.

[19] Devesa J, Reimunde P, Devesa P, Barberá M, Arce V (2013). Growth hormone (GH) and brain trauma. Horm Behav.

[20] Aberg DN, Lind J, Isgaard J, Georg Kuhn H (2010). Peripheral growth hormone induces cell proliferation in the intact adult rat brain. Growth Horm IGF Res.

[21] Christophidis LJ, Gorba T, Gustavsson M, Williams CE, Werther GA, Russo VC, Scheepens A (2009). Growth hormone receptor immunoreactivity is increased in the subventricular zone of juvenile rat brain after focal ischemia: a potential role for growth hormone in injury-induced neurogenesis. Growth Horm IGF Res.

[22] Scheepens A, Sirimanne ES, Breier BH, Clark RG, Gluckman PD, Williams CE (2001). Growth hormone as a neuronal rescue factor during recovery from CNS injury. Neuroscience.

[23] Devesa P, Reimunde P, Gallego R, Devesa J, Arce V (2011). Growth hormone (GH) treatment may cooperate with locally-produced GH in increasing the proliferative response of hippocampal progenitors to kainate-induced injury. Brain Inj.

[24] Heredia M, Fuente A, Criado J, Yajeya J, Devesa J, Riolobos AS (2013). Early growth hormone (GH) treatment promotes relevant motor functional improvement after severe frontal cortex lesion in adult rats. Behav Brain Res.

[25] Argetsinger LS, Carter-Su C (1996). Mechanism of signaling by growth hormone receptor. Physiol Rev.

[26] Kopchick JJ, Andry JM (2000). Growth hormone (GH), GH receptor and signal transduction. Mol Genet Metab.

[27] Costoya JA, Finidori J, Moutoussamy S, Señaris R, Devesa J, Arce VM (1999). Activation of growth hormone receptor delivers an antiapoptotic signal: evidence for a role of Akt in this pathway. Endocrinology.

[28] Hayashi AA, Proud CG (2007). The rapid activation of protein synthesis by growth hormone requires signaling through mTOR. Am J Physiol Endocrinol Metab.

[29] Dunlop EA, Tee AR (2009). Mammalian target of rapamycin complex 1: signalling inputs, substrates and feedback mechanisms. Cell Signal.

[30] Dowling RJ, Topisirovic I, Fonseca BD, Sonenberg N (1804). Dissecting the role of mTOR: lessons from mTOR inhibitors. Biochim Biophys Acta.

[31] Barca O, Ferré S, Seoane M, Prieto JM, Lema M, Señarís R, Arce VM (2003). Interferon beta promotes survival in primary astrocytes through phosphatidylinositol 3-kinase. J Neuroimmunol.

[32] Barca O, Seoane M, Ferré S, Prieto JM, Lema M, Señarís R, Arce VM (2007). Mechanisms of interferon-beta-induced survival in fetal and neonatal primary astrocytes. Neuroimmunomodulation.

[33] Ling L, Zhu T, Lobie PE (2003). Src-CrkII-C3G-dependent activation of Rap1 switches growth hormone-stimulated p44/42 MAP kinase and JNK/SAPK activities. J Biol Chem.

[34] Tripathi A, Sodhi A (2009). Growth hormone-induced production of cytokines in murine peritoneal macrophages in vitro: role of JAK/STAT, PI3K, PKC and MAP kinases. Immunobiology.

[35] Arce VM, Devesa P, Devesa J (2013). Role of growth hormone (GH) in the treatment of neural diseases: from neuroprotection to neural repair. Neurosci Res.

[36] Gustafson K, Hagberg H, Bengtsson BA, Brantsing C, Isgaard J (1999). Possible protective role of growth hormone in hypoxia-ischemia in neonatal rats. Pediatr Res.

[37] Blackmore DG, Golmohammadi MG, Large B, Waters MJ, Rietze RL (2009). Exercise increases neural stem cell number in a growth hormone-dependent manner, augmenting the regenerative response in aged mice. Stem Cells.

[38] Blackmore DG, Vukovic J, Waters MJ, Bartlett PF (2012). GH mediates exercise- dependent activation of SVZ neural precursor cells in aged mice. PLoS One.

[39] Blackmore DG, Reynolds BA, Golmohammadi MG, Large B, Aguilar RM, Haro L, Waters MJ, Rietze RL (2012). Growth hormone responsive neural precursor cells reside within the adult mammalian brain. Sci Rep.

[40] Pathipati P, Gorba T, Scheepens A, Goffin V, Sun Y, Fraser M (2011). Growth hormone and prolactin regulate human neural stem cell regenerative activity. Neuroscience.

[41] McLenachan S, Lum MG, Waters MJ, Turnley AM (2009). Growth hormone promotes proliferation of adult neurosphere cultures. Growth Horm IGF Res.

[42] Regalado-Santiago C, López-Meraz ML, Santiago-García J, Fernández- Pomares C, Juárez-Aguilar E (2013). Growth hormone (GH) is a survival rather than a proliferative factor for embryonic striatal neural precursor cells. Growth Horm IGF Res.

[43] Paliouras GN, Hamilton LK, Aumont A, Joppé SE, Barnabé-Heider F, Fernandes KJ (2012). Mammalian target of rapamycin signaling is a key regulator of the transit-amplifying progenitor pool in the adult and aging forebrain. J Neurosci.

[44] Zoncu R, Efeyan A, Sabatini DM (2011). mTOR: from growth signal integration to cancer, diabetes and ageing. Nat Rev Mol Cell Biol.

[45] Sarbassov DD, Guertin DA, Ali SM, Sabatini DM (2005). Phosphorylation and regulation of Akt/PKB by the rictor-mTOR complex. Science.

[46] Davis RJ (2000). Signal transduction by the JNK group of MAP kinases. Cell.

[47] Yamauchi J, Miyamoto Y, Sanbe A, Tanoue A (2006). JNK phosphorylation of paxillin, acting through the Rac1 and Cdc42 signaling cascade, mediates neurite extension in N1E–115 cells. Exp Cell Res.

[48] Wang X, Fu S, Wang Y, Yu P, Hu J, Gu W, Xu XM, Lu P (2007). Interleukin-1beta mediates proliferation and differentiation of multipotent neural precursor cells through the activation of SAPK/JNK pathway. Mol Cell Neurosci.

[49] Hong YK, Lee NG, Lee MJ, Park MS, Choi G, Suh YS, Han SY, Hwang S, Jeong G, Cho KS (2009). dXNP/DATRX increases apoptosis via the JNK and dFOXO pathway in Drosophila neurons. Biochem Biophys Res Commun.

[50] Qu C, Li W, Shao Q, Dwyer T, Huang H, Yang T, Liu G (2012). c-Jun N-terminal kinase 1 (JNK1) is required for coordination of netrin signaling in axon guidance. J Biol Chem.

[51] Zhu T, Goh EL, LeRoith D, Lobie PE (1998). Growth hormone stimulates the formation of a multiprotein signaling complex involving p130(Cas) and CrkII. Resultant activation of c-Jun N-terminal kinase/stress-activated protein kinase (JNK/SAPK). J Biol Chem.

[52] Minoia M, Gentilin E, Molè D, Rossi M, Filieri C, Tagliati F, Baroni A, Ambrosio MR, Degli Uberti E, Zatelli MC (2012). Growth hormone receptor blockade inhibits growth hormone-induced chemoresistance by restoring cytotoxic-induced apoptosis in breast cancer cells independently of estrogen receptor expression. J Clin Endocrinol Metab.

[53] Turnley AM, Faux CH, Rietze RL, Coonan JR, Bartlett PF (2002). Suppressor of cytokine signaling 2 regulates neuronal differentiation by inhibiting growth hormone signaling. Nat Neurosci.

[54] Donahue CP, Jensen RV, Ochiishi T, Eisenstein I, Zhao M, Shors T, Kosik KS (2002). Transcriptional profiling reveals regulated genes in the hippocampus during memory formation. Hippocampus.

[55] Donahue CP, Kosik KS, Shors TJ (2006). Growth hormone is produced within the hippocampus where it responds to age, sex, and stress. Proc Natl Acad Sci U S A.

[56] Sun LY, Al-Regaiey K, Masternak MM, Wang J, Bartke A (2005). Local expression of GH and IGF-1 in the hippocampus of GH-deficient long-lived mice. Neurobiol Aging.

[57] Agasse F, Roger M, Coronas V (2004). Neurogenic and intact or apoptotic non- neurogenic areas of adult brain release diffusible molecules that differentially modulate the development of subventricular zone cell cultures. Eur J Neurosci.

